# Exploring the relative stability of automated infrared pupillometry parameters under sedative and analgesic exposure after cardiac surgery

**DOI:** 10.1186/s12871-026-03889-7

**Published:** 2026-05-18

**Authors:** Saiko Okamoto, Hideaki Sakuramoto, Sayaka Hosoi, Kanji Matsuzaki, Akira Ouchi, Nobutake Shimojo, Yoshiaki Inoue

**Affiliations:** 1 Department of Emergency and Critical Care Medicine, Doctor of Medicine Program, Graduate School of Comprehensive Human Sciences, University of Tsukuba, Ibaraki, Japan; 2 Department of Nursing, Hitachi General Hospital, Ibaraki, Japan; 3 Division of Faculty Development, Kindai University, Osaka, Japan; 4 Department of Cardiovascular Surgery, Hitachi General Hospital, Ibaraki, Japan; 5 Department of Adult Health Nursing, College of Nursing, Ibaraki Christian University, Ibaraki, Japan; 6 Department of Emergency and Critical Care Medicine, Faculty of Medicine, University of Tsukuba, Ibaraki, Japan

**Keywords:** Automated infrared pupillometry, Brainstem reflex, Postoperative ICU, Sedatives and analgesics, Cardiovascular surgery

## Abstract

**Background:**

Automated infrared pupillometry (AIP) provides a quantitative, non-invasive, and reproducible method for assessing the pupillary light reflex, which is mediated by brainstem pathways. Although AIP has been increasingly used in critical care settings, interpretation of pupillary parameters may be confounded by the administration of sedatives and analgesics. However, the influence of these medications on individual AIP parameters in postoperative intensive care unit patients remains insufficiently characterized. This proof-of-concept pilot study aimed to identify pupillometric parameters that remain relatively stable under routine sedative and analgesic exposure following elective cardiovascular surgery.

**Methods:**

This prospective observational study included 10 patients admitted to the coronary care unit (CCU) after elective open-heart surgery. AIP parameters and bispectral index (BIS) values were measured postoperatively at predefined time points from CCU admission to extubation. Temporal changes in each parameter were analyzed descriptively using within-patient comparisons over the postoperative course.

**Results:**

The Neurological Pupil Index (NPi) and latency (LAT) showed minimal temporal variation throughout the observation period. In contrast, BIS values increased progressively after surgery, accompanied by gradual increases in constriction percentage (CH), mean contraction velocity (CV), and maximum contraction velocity, whereas mean dilation velocity remained relatively unchanged over time. When comparing predefined postoperative time points immediately after CCU admission and after extubation, only CH showed an increase (median [interquartile range]: 0.45 [0.38–0.52] vs. 0.68 [0.60–0.73]; *p* = 0.02).

**Conclusions:**

In this small postoperative cohort, NPi and LAT were relatively more stable than other pupillometric parameters under routine sedative and analgesic exposure. However, given the limited sample size and the absence of neurological complications, these findings should be interpreted as descriptive and hypothesis-generating, and no conclusions can be drawn regarding clinical utility for neurological monitoring or prognostication. In contrast, CH and CV require cautious interpretation, as they may be influenced by pharmacological agents rather than by the underlying neurological status.

## Background

Pupillary diameter is a clinically relevant physiological parameter that reflects autonomic nervous system activity and brainstem-mediated reflex pathways. The pupil is regulated by the sympathetic and parasympathetic pathways, with its dynamics influenced by various factors, including ambient light, emotional state, and level of consciousness [1]. The pupillary light reflex is primarily mediated by brainstem pathways, and changes in pupillary dynamics can reflect alterations in autonomic and brainstem function. In critical care settings, particularly during the postoperative period, quantitative assessment of pupillary dynamics provides objective information on brainstem-mediated pupillary reflexes under perioperative pharmacological exposure. However, conventional manual assessments of pupil size are inherently subjective and often lack reproducibility [2]. In contrast, automated infrared pupillometry (AIP) provides an objective, quantitative, and reproducible method for evaluating pupillary parameters, such as diameter, constriction velocity, latency, and the Neurological Pupil index (NPi) [3]. The recent introduction of the NeurOptics PLR-200™ pupillometer has enhanced the accessibility of this technology, facilitating the evaluation of pupil diameter even in acute care environments, such as the intensive care unit (ICU) [4].

Accordingly, AIP enables standardized quantification of brainstem-mediated pupillary reflexes under clinical conditions in which patient cooperation is limited [5]. AIP has proven particularly valuable in neurocritical care, where it enables early detection of changes in intracranial pressure and has been used in studies focused on neurological outcome assessment in conditions, such as traumatic brain injury, subarachnoid hemorrhage, and post-cardiac arrest syndrome [6–8]. Among these parameters, NPi has demonstrated promise in predicting poor neurological outcomes and early signs of cerebral herniation [9]. However, these applications have largely focused on outcome prediction in neurocritical populations, and their direct applicability to routine postoperative ICU settings remains uncertain.

In postoperative ICU patients, sedatives and analgesics are routinely administered and may influence pupillary reflex dynamics. Although pupillometry has been explored in relation to sedation assessment, its interpretation in the ICU is complex and may be confounded by factors beyond pharmacological exposure alone. In recent years, significant advancements in ICU treatment and monitoring technologies, have enable more comprehensive tracking of physiological changes. However, indicators for evaluating patient arousal and comfort in the ICU remain limited. Currently, sedation monitoring tools, such as the bispectral index (BIS) and Richmond Agitation-Sedation Scale (RASS) are widely used; however, these tools are associated with limitations. BIS primarily reflects the hypnotic component of general anesthesia rather than sedation per se and is susceptible to interference from electromyography (EMG) signals, neuromuscular blockade, or electrical noise [10], while the RASS is inherently subjective [11]. In this context, AIP offers reproducible physiological measurements; however its parameters should be interpreted as measures of brainstem-mediated pupillary reflexes rather than direct indices of sedation depth or global brain function. Although the effects of anesthetics on pupillary responses have been studied intraoperatively [12], only a few studies have examined these effects in postoperative ICU patients. Critically ill patients are often administered sedatives and analgesics. However, the specific influence of these medications on AIP parameters remains poorly understood. In particular, it is unclear which pupillometric parameters are relatively preserved and which are more susceptible to pharmacological modulation in the postoperative ICU setting.

Therefore, the primary objective of this prospective proof-of-concept pilot study was to characterize which automated pupillometry parameters demonstrated relative stability under routine postoperative sedative and analgesic exposure in postoperative ICU patients. This study was not designed to evaluate neurological outcomes, prognostication, or to establish clinical utility for neurological monitoring; rather, it provides descriptive, hypothesis-generating observations intended to inform the design of future investigations.

## Methods

### Study design and participants

This prospective, single-center, proof-of-concept observational study was conducted between October 2023 and February 2024 in the coronary care unit (CCU) of a general hospital in Japan, where patients undergoing elective open-heart surgery receive postoperative care. Patients were enrolled consecutively during a predefined, time-limited pilot phase, which accounts for the small number of included participants.

Eligible participants were adult patients who underwent elective open-heart surgery and were admitted to the CCU for postoperative care. This study was designed to explore the stability of automated pupillometry parameters under routine postoperative sedation and analgesia, rather than to evaluate neurological outcomes or prognostication. All included patients were extubated within 24 h after surgery. The exclusion criteria were as follows: patients who underwent emergency surgery, patients with impaired consciousness due to pre-existing CNS disorders at the time of admission, postoperative patients with ophthalmologic diseases, and patients with communication disorders, such as severe dementia or active psychiatric disorders that could preclude reliable clinical assessment.

### Data collection

Data were collected from each patient’s medical records at the time of CCU admission and throughout their stay. All clinical and physiological data were extracted from standardized electronic medical records and were matched across predefined postoperative time points within each individual patient. Baseline characteristics included age, sex, Acute Physiology and Chronic Health Evaluation (APACHE) II score [13], duration of CCU stay, duration of anesthesia, use of cardiopulmonary bypass, duration of mechanical ventilation, and exposure to sedatives and analgesics administered as part of routine postoperative care. Sedative and analgesic use (including propofol, dexmedetomidine, and fentanyl) was recorded descriptively and summarized by the number of patients receiving each agent, as shown in Table 2.

Detailed dose–response relationships and sedation depth assessments (e.g., RASS-based comparisons) were not systematically collected or analyzed, as the present study was not designed to evaluate sedation management or pharmacodynamic effects. The APACHE II score, a widely utilized tool for the evaluation of physiological severity of illness in ICUs, was calculated using physiological variables, age, and chronic health conditions assessed during the first 24 h of ICU admission [14].

### AIP

The pupillary light reflex was measured using a NeurOptics PLR-200™ portable infrared pupillometer (PLR-200™; NeurOptics, Irvine, CA, USA). AIP provides quantitative assessment of the brainstem-mediated pupillary light reflex under standardized conditions. The following AIP parameters were extracted from the pupillary light reflex and recorded by the instrument: maximum pupil diameter (MAX, mm), minimum pupil diameter (MIN, mm), constriction percentage (CH, %), latency (LAT, s), mean contraction velocity (CV, mm/s), maximum contraction velocity (MCV, mm/s), and mean dilation velocity (DV, mm/s). CH was defined as the percentage reduction in pupil diameter relative to baseline diameter following a standardized light stimulus. LAT was defined as the time (in seconds) from the onset of the light stimulus to the onset of pupillary constriction. The pupillometer also calculated the NPi, a unique scalar index with values ranging from 0 to 5, which reflects the overall pattern of pupillary response compared with a normative reference model, incorporating multiple pupillary response variables, including constriction and dilation characteristics.

The AIP system provides objective and reproducible measurements of pupillary reflex dynamics, but in the present study these parameters were analyzed descriptively, without attempting to infer neurological outcomes or prognostication. Previous studies have demonstrated the use of AIP in neurocritical care populations, including patients with traumatic brain injury, post–cardiac arrest syndrome, and stroke [4, 15, 16]. However, these applications differ from the present postoperative ICU setting and were not the focus of this pilot investigation.

**Fig. 1:**
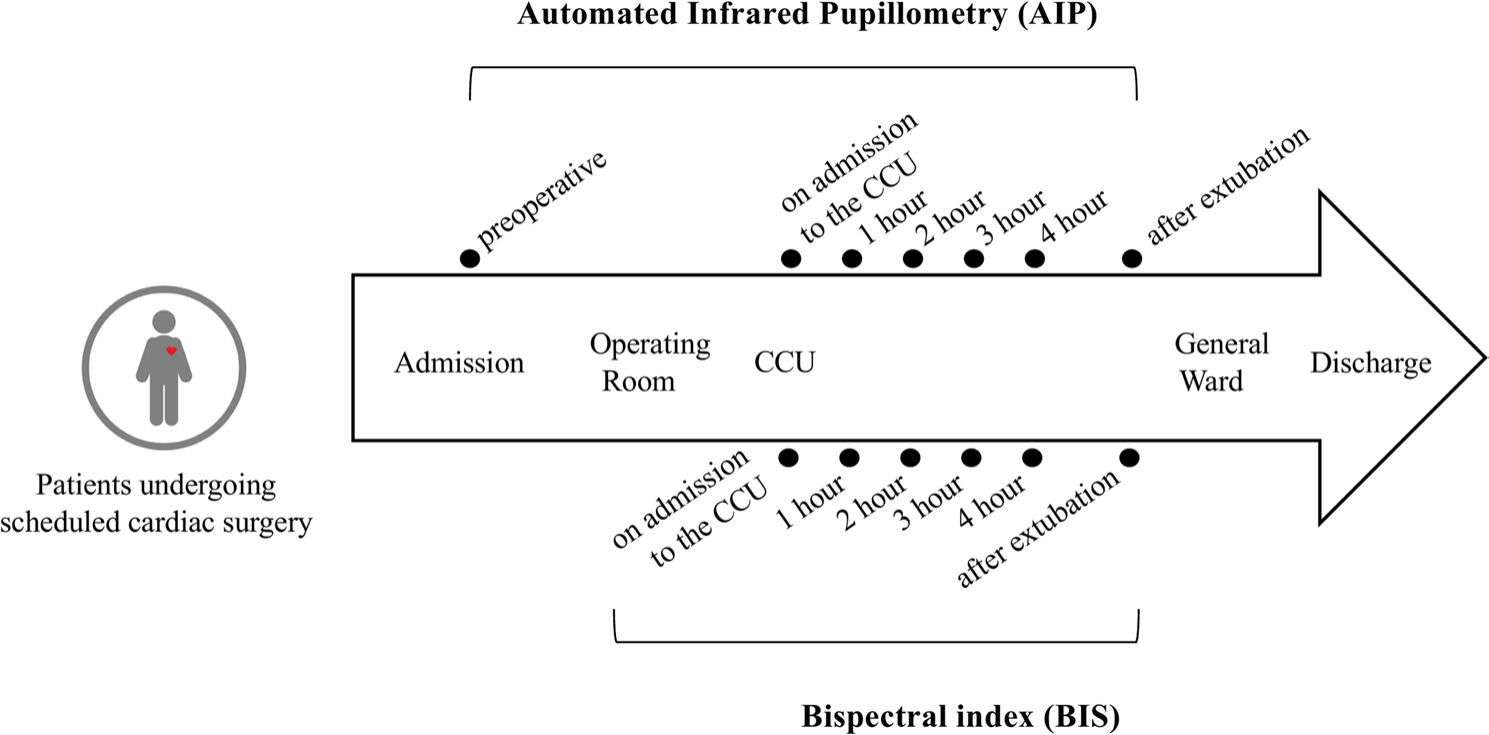
Timeline of automated infrared pupillometry and bispectral index monitoring in patients undergoing cardiac surgery

The timing of pupillometric measurements is outlined in Fig. 1: before surgery (baseline), immediately after CCU admission, and 1, 2, 3, and 4 h postoperatively, as well as after extubation (within 24 h). Baseline values (before surgery) were used as reference points, normalized to 1, and the rate of change from baseline was calculated for each subsequent time point. This approach was adopted to characterize relative temporal changes in pupillometric parameters rather than to establish diagnostic thresholds.

### BIS

The BIS is a physiological index derived from a proprietary algorithm that analyzes frontal electroencephalographic (EEG) signals reflecting cortical activity, with potential influence from EMG activity, particularly from the corrugator supercilii muscle. This noninvasive technology generates an index value ranging from 0 (isoelectric EEG) to 100 (fully awake state) using electrodes placed on the frontal and temporal areas of the scalp. BIS was originally developed to quantify the hypnotic component of general anesthesia, rather than to assess sedation depth per se. A BIS value of 40–60 is typically associated with general anesthesia, while higher values may be observed during lighter levels of hypnosis.

BIS values were continuously monitored using a dedicated monitoring system (BIS Vista Monitoring System; Medtronic, Dublin, Ireland) during the perioperative period. Although BIS has been applied in ICU settings, its interpretation in sedated critically ill patients remains limited, as it primarily reflects cortical hypnotic activity and may be influenced by EMG interference, neuromuscular blockade, and electrical noise. Accordingly, in the present study, BIS was used as a reference physiological measure rather than as a validated index of sedation depth or neurological status. BIS measurements were recorded at the same time points as pupillometry: immediately after admission to the CCU and 1, 2, 3, and 4 h postoperatively (Fig. 1). No BIS-based sedation targets or outcome predictions were defined in this study. Neuromuscular blocking agents were administered intraoperatively according to routine general anesthesia practice and were discontinued before transfer to the CCU. No neuromuscular blocking agents were administered during the postoperative CCU period, and all pupillometry and BIS measurements were performed after cessation of neuromuscular blockade.

### Ethical considerations

This study was approved by the Ethics Committees of the University of Tsukuba Hospital (Notification No. 1832) and Hitachi General Hospital (Approval No. 2022-38) and was registered with the University Hospital Medical Information Network (UMIN; ID: 000049165). This study was designed and conducted as a prospective observational study. The study adhered to the principles of the Declaration of Helsinki, and all methods were performed in accordance with the relevant ethical guidelines and regulations. The STROBE (Strengthening the Reporting of Observational Studies in Epidemiology) guidelines were followed as a reporting framework for observational research and were not intended to indicate the study design. As this was a prospective observational study, written informed consent was obtained from all participants upon hospital admission, following a detailed explanation of the study.

### Statistical analysis

Data are presented as mean ± standard deviation, median with interquartile range (IQR), or frequency with percentage, as appropriate. Temporal changes in AIP parameters and BIS values were analyzed descriptively to characterize postoperative trends from CCU admission to the time of extubation. The analyses focused on within-patient comparisons over time rather than on comparisons between predefined sedation levels. Comparisons of AIP parameters at different time points were conducted using the Wilcoxon signed-rank test, based on paired measurements obtained immediately postoperatively and after extubation within the same patients. This nonparametric approach was selected given the small sample size and the exploratory nature of the study. In the graphical representations, connected lines indicate values from the same individual. Given the proof-of-concept and exploratory design of this study, no formal sample size calculation was performed, and the analyses were intended to be descriptive rather than inferential. All statistical analyses were performed using SPSS version 29.0 (SPSS Inc., Chicago, IL, USA). Given the exploratory nature of this pilot study, p-values were calculated and are reported for descriptive purposes only and should be interpreted with caution.

## Results

### Characteristics of study participants

**Fig. 2:**
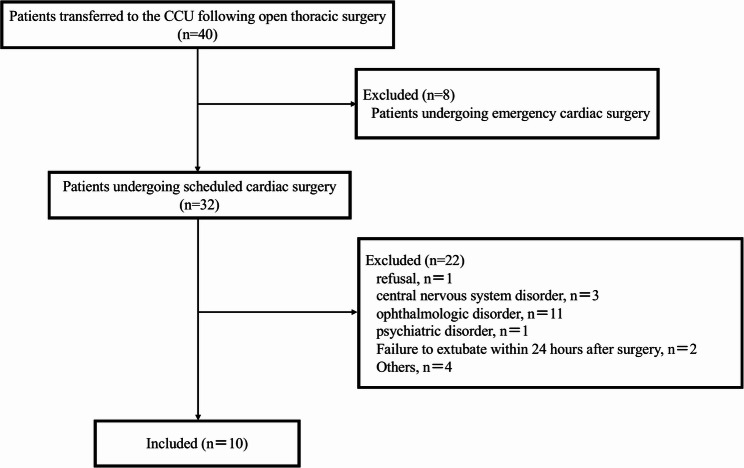
Flowchart of patients in the study cohort

During the study period, 40 patients underwent open-heart surgery and were admitted to the CCU. After screening scheduled cases and applying inclusion and exclusion criteria, 10 patients were included in this prospective proof-of-concept pilot study (Fig. 2).

Baseline demographic and clinical characteristics are summarized in Table 1. The median age was 69 years (IQR, 48–72 years), and eight patients (80%) were men. The median APACHE II score was 26 (IQR, 23–28). Surgical procedures included valve replacement (*n* = 4), combined CABG and valve replacement (*n* = 1), isolated CABG (*n* = 3), and other procedures (*n* = 2). The median CCU stay was 6 days (IQR, 1–33 days).

**Table 1:** Baseline demographic and clinical characteristics of the study participants

	Total *N* = 10
Age, years	69 [48, 72]
Male sex, n	8
APACHE II score	26 [23, 28]
ASA-PS, n	
Class II	3
Class Ⅲ	6
Class Ⅳ	1
Surgical Technique, n	
coronary artery bypass grafting	3
valve replacement	4
coronary artery bypass grafting, and valve replacement	1
other	2
Length of CCU stay, days	6 [1, 33]
Anesthesia time, minute	420 [349, 483]
Heart-lung Machine, n	7
time of a heart-lung machine, minute	150 [110, 190]
Mechanical ventilation (CCU), minute	248 [230, 859]
Anesthetic agents	
SEV%, n	9
Fentanyl, µg/kg	8.6 [7.4, 12]
remifentanil, µg/kg	133.1 [109.1, 165.3]
Propofol, mg/kg/anesthesia time	3.7 [1.6, 4.3]
Rocuronium, mg/kg	2.1 [1.8, 3.2]

Data are presented as median (interquartile range) or number (%), as appropriate. Sedative and analgesic agents administered during the postoperative CCU period are summarized separately in Table 2

*Abbreviations*: *APACHE II* Acute Physiology and Chronic Health Evaluation II, *ASA-PS* American Society of Anesthesiologists Physical Status, *CCU* Coronary Care Unit, *SEV* sevoflurane

Exposure to anesthetic, sedative, and analgesic agents is summarized in Table 2. During surgery, sevoflurane was administered to nine patients (90%). Postoperative sedative and analgesic management in the CCU followed routine clinical practice. On postoperative day 0, propofol was administered to eight patients, dexmedetomidine to five patients, and fentanyl to one patient; exposure decreased on postoperative day 1 (Table 2).

**Table 2:** Exposure to sedatives and analgesics during the postoperative coronary care unit period

	Total *N* = 10
Analgesia and sedation on the day of surgery	
Propofol, n (%)	8 (80)
Propofol, mg/kg/day	2.3 [0.5, 6.5]
Dexmedetomidine, n (%)	5 (50)
Dexmedetomidine, µg/kg/day	1.6 [0.9, 2.4]
Fentanyl, n (%)	1 (10)
Fentanyl, µg/kg/day	25.3
Analgesia and sedation on postoperative day 1	
Propofol, n (%)	1 (10)
Propofol, mg/kg/day	32.5
Dexmedetomidine, n (%)	4 (40)
Dexmedetomidine, µg/kg/day	0.6 [0.3, 2.5]
Fentanyl, n (%)	1 (10)
Fentanyl, µg/kg/day	57.7

Values indicate the number (%) of patients receiving each sedative or analgesic agent. Medication exposure is reported separately for the day of surgery (postoperative day 0) and postoperative day 1. Dose values are presented as median (interquartile range) and were calculated only for patients who received the corresponding medication. All medications were administered according to routine clinical practice, and dosing was not standardized

*Abbreviations*: *CCU* Coronary Care Unit

### Postoperative changes in BIS values and AIP parameters after open-heart surgery

**Fig. 3:**
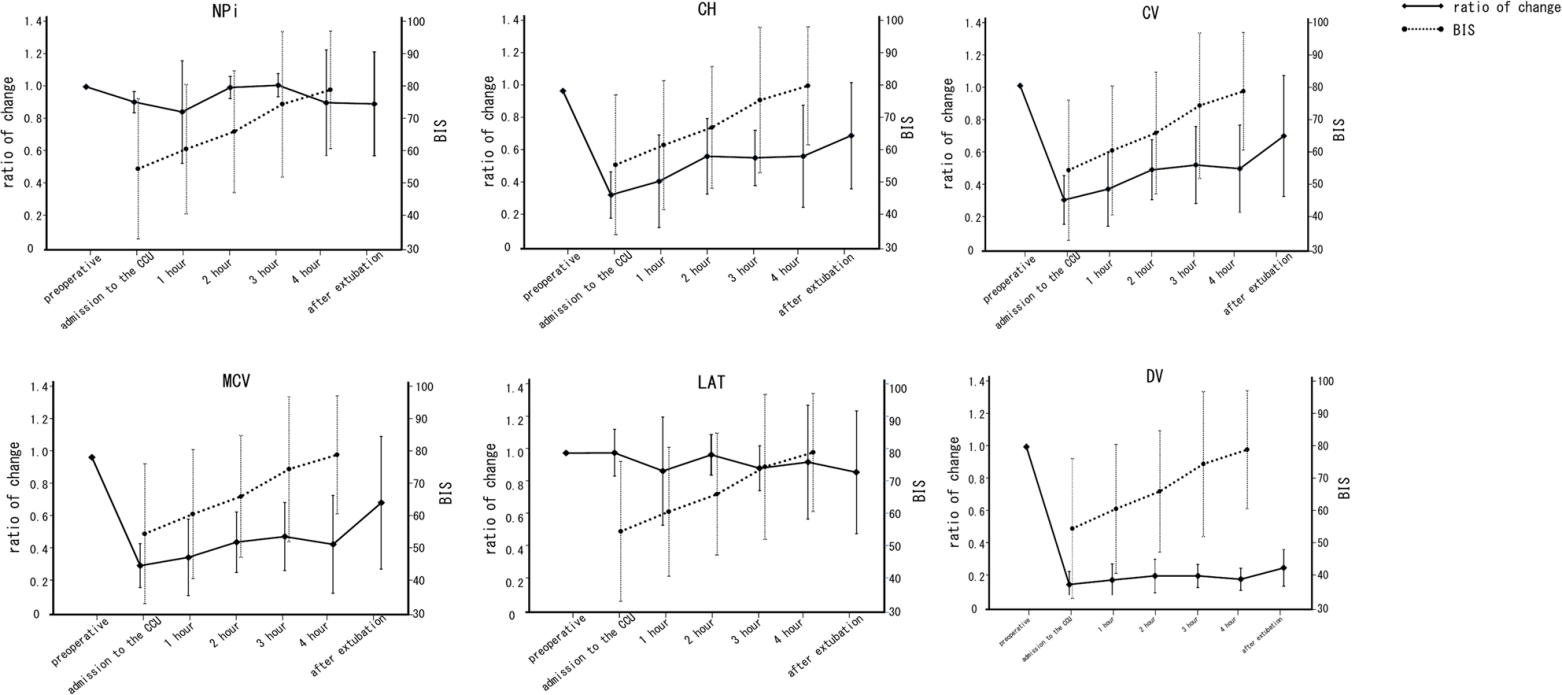
Postoperative time course of bispectral index values and automated infrared pupillometry parameters. This figure shows temporal changes in BIS values and the ratio of change from baseline in AIP parameters measured at predefined postoperative time points after admission to the CCU. Each panel represents a different AIP parameter (NPi, CH, CV, MCV, LAT, or DV). Solid lines indicate individual patient trajectories of AIP parameters, and dotted lines indicate corresponding BIS values. Abbreviations: BIS, Bispectral Index; AIP, automated infrared pupillometry; CCU, Coronary Care Unit; NPi, Neurological Pupil Index; CH, constriction percentage; CV, mean constriction velocity; MCV, maximum constriction velocity; LAT, latency; DV, mean dilation velocity

**Fig. 4:**
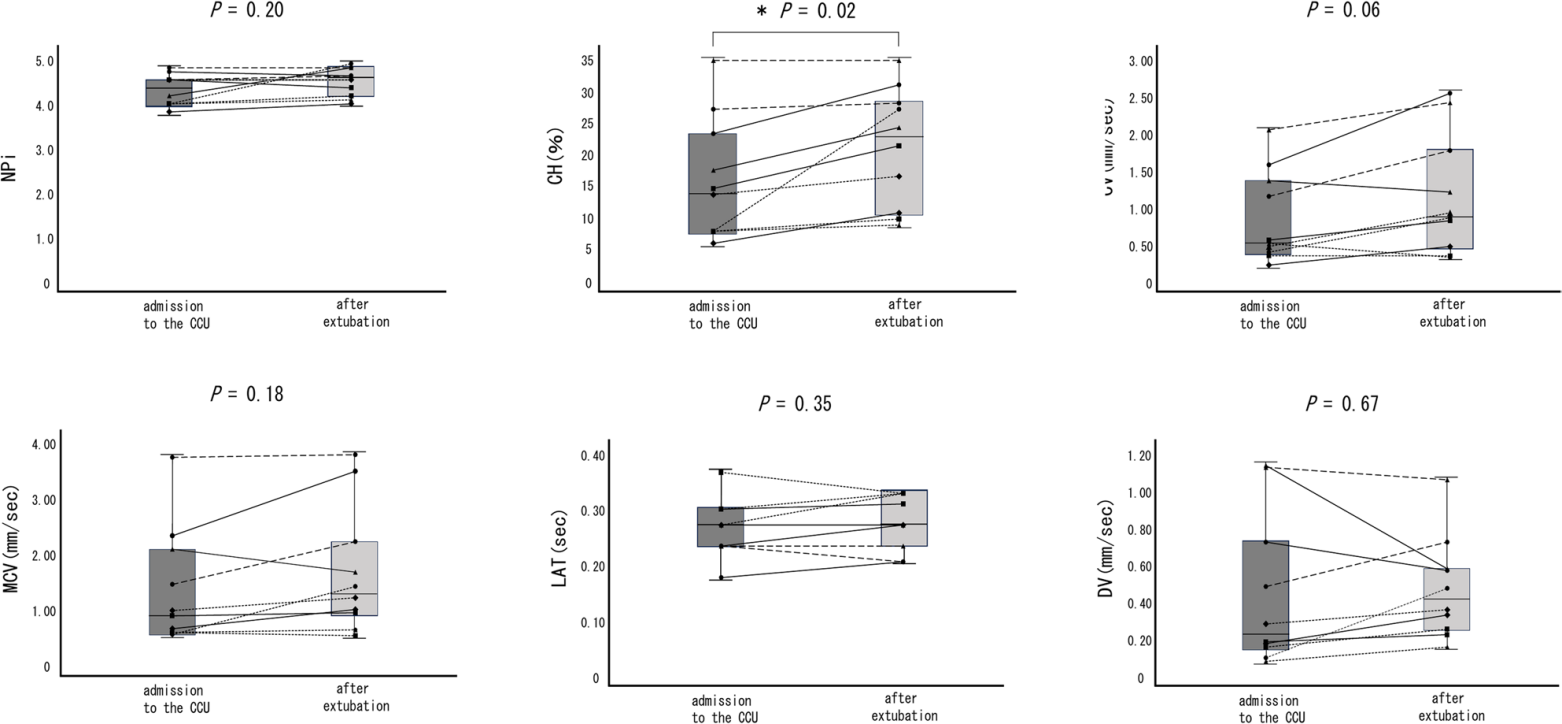
Comparison of automated infrared pupillometry parameters between predefined postoperative time points. Each panel shows changes in AIP parameters between measurements obtained immediately after admission to the CCU and after extubation. Lines connect paired values from the same individual. Boxplots represent the median and interquartile range (IQR). *P* values were calculated using the Wilcoxon signed-rank test and are reported for descriptive purposes. Abbreviations: AIP, automated infrared pupillometry; CCU, Coronary Care Unit; NPi, Neurological Pupil Index; CH, constriction percentage; CV, mean constriction velocity; MCV, maximum constriction velocity; LAT, latency; DV, mean dilation velocity

Postoperative changes in BIS and AIP parameters are shown in Fig. 3. Over the postoperative course, NPi and LAT showed minimal temporal variation, whereas CH, CV, and MCV exhibited greater variability. Comparisons between CCU admission and post-extubation are presented in Fig. 4. Among the evaluated parameters, only CH showed an increase between these two time points (median [IQR]: 0.45 [0.38–0.52] vs. 0.68 [0.60–0.73]), whereas CV, MCV, DV, NPi, and LAT showed no consistent changes. Given the exploratory nature and small sample size of this study, p-values are reported for descriptive purposes only.

Overall, these observations indicate that CH exhibited a measurable postoperative change, whereas NPi and LAT remained relatively stable across the observed postoperative period in this cohort.

## Discussion

In this prospective proof-of-concept pilot study, we found that pupillometric parameters differed in their susceptibility to sedative and analgesic exposure, with NPi and LAT remaining relatively stable, whereas CH, CV, MCV, and DV exhibited variability in postoperative ICU patients. These observations were based on within-patient postoperative comparisons and reflect descriptive temporal patterns rather than causal effects of individual drugs. Although automated pupillometry is increasingly used in ICU settings, interpretation of pupillary dynamics may be confounded by perioperative pharmacological exposure. Accordingly, these findings highlight that, within this small postoperative cohort, some pupillometric parameters demonstrated relative stability, without supporting conclusions regarding clinical utility or decision thresholds during the perioperative period [4, 6–8].

Our findings are broadly consistent with prior research examining pupillary light reflex parameters measured by AIP in various clinical contexts. Notably, NPi and LAT showed minimal change over the postoperative course despite exposure to sedatives and analgesics [17]. This observation indicates relative stability of these parameters within the present cohort, rather than suggesting that they directly reflect preserved neurological function under sedation. LAT represents the initiation of the pupillary light reflex arc, which is primarily mediated by afferent pathways and brainstem circuits. Such brainstem-mediated processes may be less susceptible to cortical depressant effects of sedatives and analgesics, although this interpretation remains speculative [18]. Similarly, NPi integrates multiple pupillary response metrics into a composite index, which may attenuate variability in individual parameters and contribute to its relative stability [19, 20].

Previous studies in neurocritical care have demonstrated the robustness of NPi and LAT in assessing brain function and, in some cases, predicting neurological outcomes [21, 22]. However, these studies focused on populations with overt neurological injury, which differs from the postoperative cohort examined in the present study. Accordingly, our findings do not have prognostic implications. Instead, they describe the relative stability of NPi and LAT under routine postoperative sedative and analgesic exposure. Because no neurological complications occurred in this cohort, the present findings do not allow the evaluation of the utility of theses parameters for detecting neurological deterioration or predicting outcomes.

In contrast, parameters such as CH and CV exhibited greater temporal variability and appeared more susceptible to postoperative pharmacological exposure. CH, which reflects the magnitude of pupillary constriction in response to light stimulation, is influenced by parasympathetic activity and iris sphincter muscle responsiveness [23]. In the present study, CH was the only AIP parameter that demonstrated a measurable change between predefined postoperative time points. Previous studies have suggested that sedatives and analgesics, including opioids and α2-agonists, can alter pupillary constriction dynamics [24, 25], supporting the observation that CH and related velocity parameters may be particularly sensitive to pharmacological modulation. Therefore, changes in these parameters should be interpreted cautiously, as they may reflect drug effects rather than intrinsic alterations in brainstem reflex function.

In summary, a major finding of this study is that automated pupillometry can objectively and quantitatively evaluate the dynamics of pupillary reflexes and may predict neurological complications with selectively appropriate parameters in postoperative ICU patients, even immediately after anesthetic recovery and under sedative and analgesic administration. However, given the small sample size, absence of neurological complications, and non-standardized sedation regimen, the results should be interpreted as descriptive and hypothesis-generating. The present study does not support conclusions regarding clinical monitoring or prognostication but highlights differential stability among pupillometric parameters under routine postoperative sedation.

This study had several limitations. First, the single-center design and small sample size (*n* = 10) the limit generalizability of the study findings. Second, the observational study design precludes causal inference between drug administration and pupillometric changes. Third, sedatives and analgesics were administered according to routine clinical practice without standardized protocols, and the effects of individual drug doses and combinations were not evaluated. In addition, measurement timing was not aligned with pharmacokinetic profiles, and other physiological factors, such as hemodynamic management, were not systematically analyzed. Finally, the exclusion of patients with neurological or ophthalmologic conditions limits applicability of the study findings to broader ICU populations. Accordingly, the present findings should not be extrapolated to neurological monitoring or outcome prediction scenarios but should be considered preliminary observations that can from the basis for future research.

## Conclusions

In this proof-of-concept pilot study of postoperative cardiac surgery patients, we observed that some automated pupillometry parameters, particularly NPi and LAT, were relatively more stable than others under routine sedative and analgesic exposure. However, given the small sample size and the absence of neurological complications, no conclusions can be drawn regarding the clinical utility of these parameters for brain monitoring or prognostication. These findings should therefore be interpreted as descriptive observations, intended to inform the design of future studies.

## Data Availability

The datasets used and/or analyzed during the current study are available from the corresponding author on reasonable request.

## Abbreviations

AIP: Automated Infrared Pupillometry
APACHE II: Acute Physiology and Chronic Health Evaluation II
ASA-PS: American Society of Anesthesiologists Physical Status
BIS: Bispectral Index
CCU: Coronary Care Unit
CH: Constriction percentage
CV: Constriction Velocity
DV: Dilation Velocity
LAT: Latency
MAX: Maximum pupil diameter
MCV: Maximum Constriction Velocity
MIN: Minimum pupil diameter
NPi: Neurological Pupil index
RASS: Richmond Agitation-Sedation Scale
SEV: Sevoflurane
